# A Thirty-Five-Minute Nap Improves Performance and Attention in the 5-m Shuttle Run Test during and outside Ramadan Observance

**DOI:** 10.3390/sports8070098

**Published:** 2020-07-11

**Authors:** Hsen Hsouna, Omar Boukhris, Khaled Trabelsi, Raouf Abdessalem, Achraf Ammar, Jordan M. Glenn, Nick Bott, Nizar Souissi, Paola Lanteri, Sergio Garbarino, Nicola Luigi Bragazzi, Hamdi Chtourou

**Affiliations:** 1 “Physical Activity, Sport and Health” Research Unit, UR18JS01, National Sport Observatory, Tunis 1003, Tunisia; hsen.hsouna92@gmail.com (H.H.); omarboukhris24@yahoo.com (O.B.); raoufabdesalem18@gmail.com (R.A.); n_souissi@yahoo.fr (N.S.); h_chtourou@yahoo.fr (H.C.); 2 High Institute of Sport and Physical Education of Sfax, University of Sfax, Sfax 3000, Tunisia; trabelsikhaled@gmail.com; 3 Research Laboratory: Education, Motricity, Sport and Health, EM2S, LR19JS01, High Institute of Sport and Physical Education of Sfax, University of Sfax, Sfax 3000, Tunisia; 4 Institute of Sport Science, Otto-von-Guericke University Magdeburg, Universitätsplatz 2, 39106 Magdeburg, Germany; Ammar.achraf@ymail.com; 5 Department of Health, Exercise Science Research Center Human Performance and Recreation, University of Arkansas, Fayetteville, AR 72701, USA; jordan@neurotrack.com; 6 Neurotrack Technologies, 399 Bradford St. Redwood City, CA 94063, USA; nick@neurotrack.com; 7 Department of Medicine, Clinical Excellence Research Center, Stanford University School of Medicine, Stanford, CA 94305, USA; 8 Neurophysiopathology Unit, Foundation IRCCS Carlo Besta Neurological Institute, 20133 Milan, Italy; paola.lanteri@istituto-besta.it; 9 Department of Neuroscience, Rehabilitation, Ophthalmology, Genetics, Maternal and Child Health (DINOGMI), University of Genoa, 16132 Genoa, Italy; sgarbarino.neuro@gmail.com; 10 Department of Health Sciences (DISSAL), Postgraduate School of Public Health, University of Genoa, 16132 Genoa, Italy; 11 Department of Mathematics and Statistics, Laboratory for Industrial and Applied Mathematics (LIAM), York University, Toronto, ON M3J 1P3, Canada

**Keywords:** performance, exercise, fasting, psychological, siesta

## Abstract

Ramadan observance is characterized by several changes in behaviors, such as food and sleep, which could affect physical and cognitive performance. The aim of the present study was to investigate the effects of a 35-min nap (N35) opportunity on physical performance during the 5-m shuttle run test (5mSRT); attention; feelings; mood states; and perceptual measures of stress, fatigue, and muscle soreness during Ramadan observance. Fourteen physically active men (22 ± 3 years, 177 ± 4 cm, 76 ± 5 kg) were tested after a no-nap condition (N0), N35 15 days before Ramadan (BR), the last 10 days of Ramadan (DR), and 20 days after Ramadan (AR). Measures included the digit cancellation test (attention estimation), the profile of mood state (POMS), and the Hooper questionnaires. After a 5-min standard warm-up, participants performed the 5mSRT (6 × 30 s with 35 s in between; best distance (BD), total distance (TD), and fatigue index (FI) were recorded), along with the rating of perceived exertion (RPE) after each test repetition. After the 5mSRT test, participants responded to the feeling scale (FS). The results showed that TD and FI during the 5mSRT were not affected by Ramadan observance. However, BD was significantly lower than DR compared to AR after N0 (∆ = −4.3 ± 1.3%; *p* < 0.01) and N35 (∆ = −2.6 ± 1.0%; *p* < 0.05). After N0, attention decreased significantly at DR in comparison with BR (*p* < 0.05) and AR (*p* < 0.001). BD and TD improved after N35 compared to N0 at BR (∆ = +4.4 ± 2.1%, *p* < 0.05 for BD and ∆ = +4.8 ± 1.6%, *p* < 0.01 for TD), DR (∆ = +7.1 ± 2.2%, *p* < 0.05 for BD and ∆ = +5.1 ± 1.6%, *p* < 0.01 for TD), and AR (∆ = +5.5 ± 1.5%, *p* < 0.01 for BD and ∆ = +5.2 ± 1.2%, *p* < 0.001 for TD). A significant increase in attention was observed after N35 in comparison with N0 at DR (*p* < 0.01) and AR (*p* < 0.01). However, no changes were found for the perception of mood states, stress, sleep, muscle soreness, and the FI during the 5mSRT. Also, N35 was better than N0 for RPE at DR (*p* < 0.05), feelings at AR (*p* < 0.05), and fatigue estimation at AR (*p* < 0.01). A 35-min nap opportunity may have beneficial effects on physical and cognitive performances before, during, and after Ramadan.

## 1. Introduction

Intermittent fasting is an obligation for healthy pubescent Muslims during the month of Ramadan (observance lasting between 29 and 30 days) [1]. During this month, it is prohibited for Muslims to eat, drink, smoke, or have sexual intercourse from dawn to sunset (fasting period) [2]. As a result, these obligations reduce diurnal and increase nocturnal social activities in mainly Muslims countries [3].

Changes in eating schedules as well as other activities (i.e., late prayer (*Taraweeh*)) have considerable effects on sleep [4,5,6,7] and food/fluid intakes [8,9]. Other changes may also be observed during this month [10,11]. For example, Chtourou et al. [3] reported changes in mood states and participant chronotype during the month of Ramadan. With these changes, there is the possibility of negative effects on physical [12,13,14,15] and cognitive [11] performances during this month.

Boukhris et al. [11] reported a significant decrease in sleep efficiency and duration during and after Ramadan compared to before the fasting month. Similarly, perception of sleep quality was reported to be lower during compared to before Ramadan [16]. In this context, it has been reported that light-sleep stage duration increases significantly during Ramadan. This may be attributed to an increase in the number of awakenings, reflecting increased nighttime metabolism due to the late *Souhour* meal (last meal before starting the day fast) [17]; it may also be attributed to increased food-seeking behaviors. Additionally, Zerguini et al. [18] suggested that poor sleep quality during Ramadan could be explained by the accommodation of late evening food intake. This reduction in sleep duration may impede physical performance as studies report that sleep reduction has negative impacts on cognitive [19,20] and physical [21,22] performance. As adequate sleep (quantity and quality) is important for maximizing cognitive and physical performance as well as physical recovery [23], recent studies suggest that the inclusion of a short nap during the daytime (i.e., between 13 h and 15 h) may help overcome these detrimental effects in performance. Previously, beneficial effects of a 30-min nap on 20-m sprint times have been reported [24]. When repeated sprint exercise was completed after a bout of sleep deprivation, Hammouda et al. [25] reported positive effects from 20-min and 90-min nap opportunities. The nap-related beneficial effects also exist after a night of normal sleep (25-min nap [26,27] and 45-min nap [28]). For example, during the 5-m shuttle run test (5mSRT), Boukhris et al. [26] and Abdessalem et al. [27] reported that a 25-min nap enhanced physical performance. In this way, Hammouda et al. [25] suggested that a nap opportunity could represent a full sleep cycle, which contains slow wave sleep (SWS) and rapid eye movement (REM) sleep stages. SWS is a known recovery period for daily metabolism. Thus, Boukhris et al. [26] reported that the beneficial effect of nap on physical performance could be explained by an improvement of alertness and reduction of sleepiness and fatigue. As the effects of Ramadan on sleep may represent a series of sleep abnormalities, the inclusion of a nap after sleep during this period may be beneficial for physical and cognitive performance.

Therefore, the aim of the present study was to investigate the effects of a 35-min nap before, during, and after Ramadan on physical and cognitive performance in physically active men. In a recent study, Hsouna et al. [29] showed that 25-min of nap opportunity had no significant effect on the 5mSRT performance and attention before and during Ramadan; a significant positive effect was reported only after Ramadan. The authors suggested that longer nap duration is required to elicit a significant effect on performance during Ramadan. Previous studies evaluating the effects of different nap durations (i.e., 25 min, 35 min, and 45 min) on physical and cognitive performance showed a more beneficial effect of nap of 35 min and 45 min durations compared to 25 min [26,28]. Therefore, we hypothesized that 35-min of nap opportunity before and during Ramadan would improve performance in both categories. We also collected mood states, subjective perception of stress, muscle soreness, fatigue, and sleep quality. We hypothesized that the 35-min nap opportunity would overcome the previously reported negative impacts of Ramadan on mood states [3] and sleep quality [16]. Finally, as Boukhris et al. [11] reported that the reductions in sleep duration during Ramadan were still present after the fasting month, we hypothesized that a 35-min nap would also be beneficial after Ramadan.

## 2. Materials and Methods

### 2.1. Participants

A minimum of eleven participants was required for inclusion in the present study. This sample size was calculated using the software G*power (version 3.1.9.2; Kiel University, Kiel, Germany, α = 0.05 and power = 0.95) and based on the effect size (= 0.46) shown by Herrera et al. [30]. Due to potential dropout, twenty participants were recruited. Five participants were excluded from the data analysis as they did not complete all required sessions (n = 3) or failed to initiate sleep (n = 2). After introducing the study and obtaining their written consent, fourteen physically active males (age: 22 ± 3 years, height: 177 ± 4 cm, body-mass: 76 ± 5 kg) participated in the present study. The participants were recruited by advertising in classes and posting notices on bulletin boards. They did not have any pathological disorders, and they regularly performed fast walking and jogging at 3 × 1-h sessions/week in the late afternoon during the study. The study was conducted according to the Declaration of Helsinki and was fully approved by the local ethics committee (CPP: 0098/2018).

### 2.2. Experimental Design

All participants were familiarized to the protocol and tests of the present study. They then participated randomly in two testing sessions: a no-nap opportunity (N0) and a 35-min nap opportunity (N35). Each testing session occurred during three separate occasions: 15 days before Ramadan (BR), the last 10 days of Ramadan (DR), and 20 days after Ramadan (AR). At least 72 h was required between successive testing sessions. During each condition, participants came to the laboratory for a period of time between 13h45 and 18h30. For the nap, participants were allotted 15 min to be accustomed to the new place of sleep in the sleep laboratory. Following the 15 min acclimation period, participants completed the 35-min nap in dark and quiet sleep rooms. After N35 and during N0, participants did not engage in physical activity but were allowed to perform leisure activities such as watching TV, playing video games, etc. in a comfortable armchair. At 17h00, participants completed the digit cancellation test (assessment of attention), 5-min of standardized warm-up, and the 5mSRT (6 × 30 s with 35 s in between). The digit cancellation test consists of deleting target numbers (i.e., numbers composed by three grouped digits) on a sheet of randomly arranged possibilities; the sum of correct responses was recorded for analysis.

During BR, DR, and AR (after the nap conditions), after the end of the 5mSRT, the Hooper questionnaire was used to estimate sleep, stress, fatigue, and muscle soreness using subjective scales ranging from “1” (very low or good) to “7” (very high or bad) [31]. During the same period, before the 5mSRT at 16h45, the profile of mood states (POMS) questionnaire was used to evaluate seven states (i.e., tension, depression, anger, vigor, fatigue, confusion, and interpersonal relationships), where participants respond to 65 adjectives on a scale ranging from “0” (not at all) to “4” (extreme) [32].

Rating of perceived exertion (RPE) was estimated after each repetition of the 5mSRT on a scale ranging from “0” (very light) to “10” (very hard) [33]. The mean score during the 5mSRT was calculated for analysis. Feelings (FS) scores were recorded after the 5mSRT on a scale ranging from “−5” (feeling very displeased) to “+5” (feeling pleasure) by responding to the question: “How are you feeling, and how good or bad did you feel?” [34].

During BR, DR, and AR, participants were instructed to record estimated quantities of all food and beverages consumed for ten days during each period of physical testing. The amount and type of food and fluid consumed were calculated using the software program Bilnut (Nutrisoft Bilnut: Food Survey Program version 2.01) and the food-composition tables of the Tunisian National Institute of Statistics (1978) by an expert nutritionist after interviewing all participants.

Furthermore, participants were allowed to bring their own food and drink and consume ad libitum.

Sleep duration, sleep quality, sleep latency, sleep efficiency, sleep disturbances, the use of sleeping medications, and daytime dysfunction were estimated using the Pittsburgh Sleep Quality Index (PSQI), containing 19 questions [35]. The total score of the PSQI ranged from “0” (no difficulty)” to “21” (severe difficulties).

The collection of the digit cancelation test, POMS, and PSQI data took place in the laboratory, while the 5mSRT, RPE, and FS data collection took place on a sport field.

During the study, participants fasted for ~15–16 h per day. During BR, DR, and AR, the temperature and relative humidity were respectively 28 °C and 50%, 32 °C and 49%, and 31 °C and 47%.

### 2.3. The 5-m Shuttle Run Test

As described by Boukhris et al. [36], participants were required to cover the maximal distance during shuttle sprints of 5 m, 10 m, 15 m, 20 m, etc, until the end of 30 s. This task was performed three times with 35 s of recovery in between.

During the test, the following parameters were calculated:

- Total distance (TD) = Sum of distances in meter covered during the 6 × 30-s shuttles
- Best distance (BD) = The higher distance in meter realized during the 6 × 30-s shuttles
- Fatigue index (FI):
  FI (%) = (((shuttle 1 + shuttle 2) − (shuttle 5 + shuttle 6))/(shuttle 1 + shuttle 2)) × 100(1)

### 2.4. Statistical Analyse

Data were presented as mean ± standard deviation (SD) and analyzed using STATISTICA 12.0 Software (Stat-Soft, Maisons-Alfort, Paris, France). Normality of the distribution was confirmed using Shapiro Wilk’s test. TD, PSQI, carbohydrates, and lipids data were normally distributed. A two-factor ANOVA (period × nap) was performed for TD, and a one-factor ANOVA (period) was performed for PSQI, carbohydrates, and lipids data. Post hoc comparisons were completed using the Bonferroni test; effect sizes were calculated as partial eta-squared (η_p_^2^). However, when the normality was not confirmed, a Friedman test was performed [37] for BD, FI, attention, FS, RPE, calorie and protein intake, Hooper indices, and POMS parameters; effect sizes were estimated by Kendall’s coefficient of concordance [38]. When significant, pairwise comparisons were also conducted using a Wilcoxon test. Significance was accepted for all analyses at the level of *p* ≤ 0.05. Due to the high number of dependent variables, only significant differences are reported.

## 3. Results

### 3.1. The 5-m Shuttle Run Test

#### 3.1.1. Total Distance

**Figure 1:**
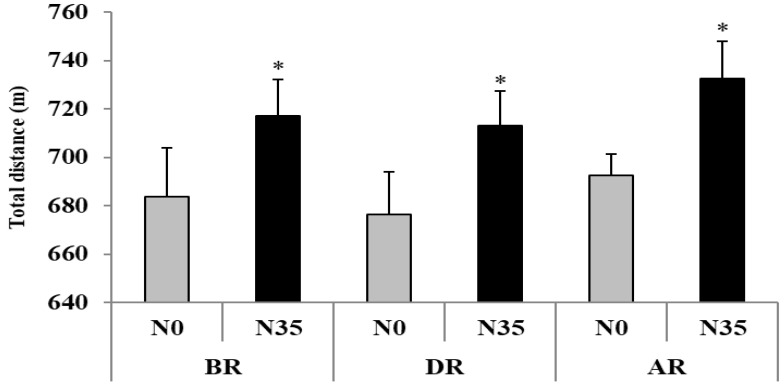
Total distance (mean ± SD) during the 5-m shuttle run test recorded before (BR), during (DR), and after (AR) Ramadan after no-nap (N0) and 35-min nap (N35) opportunities. * Significant difference in comparison with N0.

The repeated measures ANOVA revealed a significant main effect of nap (F = 19.29; *p* < 0.001; η_p_^2^ = 0.59) (Figure 1). The Bonferroni post hoc test revealed a significant increase of TD after N35 compared with N0 at BR (∆ = +4.8 ± 1.6%; *p* < 0.01), DR (∆ = +5.1 ± 1.6%; *p* < 0.01), and AR (∆ = +5.2 ± 1.2%; *p* < 0.001).

#### 3.1.2. Best Distance

**Figure 2:**
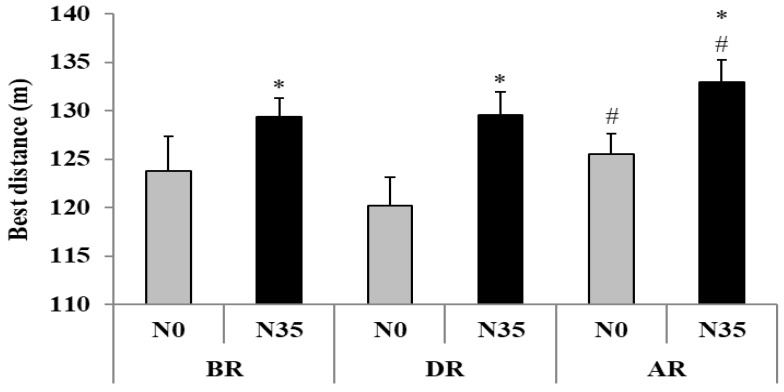
Best distance (mean ± SD) during the 5-m shuttle run test recorded before (BR), during (DR), and after (AR) Ramadan after no-nap (N0) and 35-min nap (N35) opportunities. * Significant difference in comparison with N0; # significant difference in comparison with DR.

The Friedman test revealed a significant main effect (test = 22.76, *p* < 0.001, Kendall’s W = 0.32) (Figure 2). Pairwise comparisons demonstrated a significant increase of BD after N35 when compared with N0 at BR (∆ = +4.4 ± 2.1%; *p* < 0.05), DR (∆ = +7.1 ± 2.2%; *p* < 0.05), and AR (∆ = +5.5 ± 1.5%; *p* < 0.01). However, BD was lower at DR in comparison with AR in N0 (∆ = +4.3 ± 1.3%; *p* < 0.01) and N35 (∆ = +2.6 ± 1.0%; *p* < 0.05).

#### 3.1.3. Fatigue Index

**Figure 3:**
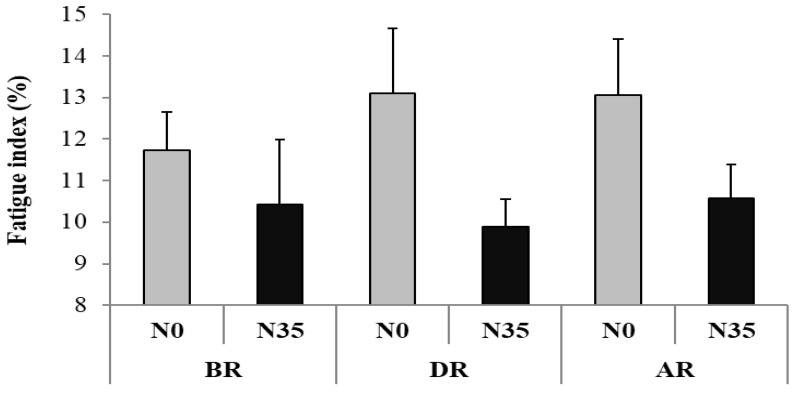
The fatigue index (mean ± SD) during the 5-m shuttle run test recorded before (BR), during (DR), and after (AR) Ramadan after no-nap (N0) and 35-min nap (N35) opportunities.

The Friedman test did not show any significant main effect (test = 9.72, *p* > 0.05, Kendall’s W = 0.13) (Figure 3).

### 3.2. Attention Scores Recorded by the Digit Cancellation Test 

Attention scores are presented in Table 1.

The Friedman test revealed a significant main effect (test = 35.49, *p* < 0.001, Kendall’s W = 0.5). The Wilcoxon post hoc test showed that attention scores were significantly lower after N0 at DR in comparison with BR (*p* < 0.05) and AR (*p* < 0.01). After N35, attention scores were higher at AR in comparison with BR (*p* < 0.01). However, pairwise comparisons revealed a significant increase in attention after N35 in comparison with N0 at DR (*p* < 0.01) and AR (*p* < 0.01) (Table 1).

### 3.3. Rating of Perceived Exertion Scores

RPE scores are presented in Table 1. The Friedman test revealed a significant main effect (test = 22.84, *p* < 0.001, Kendall’s W = 0.32). Pairwise comparisons revealed a significant decrease of RPE scores recorded after N0 at AR when compared with BR *(p* < 0.01) and DR *(p* < 0.01). Also, RPE scores were significantly lower after N35 in comparison with N0 at DR *(p* < 0.05) (Table 1).

### 3.4. Feelings Scale

Feelings scores are presented in Table 1. The Friedman test revealed a significant main effect (test = 19.68, *p* < 0.01, Kendall’s W = 0.28). The Wilcoxon post hoc test showed that feelings scores significantly decreased at DR in comparison with BR after N0 (*p* < 0.05) and after N35 (*p* < 0.05). After N35, feelings scores were significantly higher at AR than DR (*p* < 0.05). Feelings scores were significantly higher at AR after N35 in comparison with N0 *(p* < 0.05) (Table 1).

### 3.5. Dietary Intake

No significant effect of periods on calories intake (test = 1.41; *p* > 0.05; Kendall’s W = 0.05), proteins (test = 0.12, *p* > 0.05, Kendall’s W = 0.004) and lipids (F = 2.4; P > 0.05; η_p_^2^ = 0.15) was reported. However, statistical analyses revealed a significant effect for carbohydrates (F = 5.02; *p* < 0.05; η_p_^2^ = 0.27) (Table 2), wherein carbohydrates were significantly higher during AR in comparison with DR.

### 3.6. The PSQI Questionnaire

PSQI parameters are presented in Table 3.

No significant effect was reported for sleep latency (test = 2.07, *p* > 0.05, Kendall’s W = 0.07), sleep efficiency (test = 3.5, *p* > 0.05, Kendall’s W = 0.12), or sleep disturbances (test = 4.66, *p* > 0.05, Kendall’s W = 0.16) (Table 3).

#### 3.6.1. Sleep Quality

A significant effect was reported (test = 14.97, *p* < 0.0005, Kendall’s W = 0.53). The sleep quality scores increased significantly at DR (*p* < 0.01) and AR (*p* < 0.05) in comparison with BR.

#### 3.6.2. Sleep Duration

There was a significant effect (test = 8.21, *p* < 0.05, Kendall’s W = 0.29) with decrease in sleep duration DR in comparison with BR (*p* < 0.05).

#### 3.6.3. Daytime Dysfunction

There was a significant effect (test = 8.43, *p* < 0.05, Kendall’s W = 0.3) with higher sleep dysfunction scores DR in comparison with BR (*p* < 0.05).

#### 3.6.4. Total PSQI Scores

The statistical analysis revealed a significant effect (F = 18.59; *p* < 0.001; η_p_^2^ = 0.59). Total PSQI scores were significantly higher in DR (*p* < 0.001) and AR (*p* < 0.05) in comparison with BR. 

### 3.7. Hooper Questionnaire Indices 

Sleep, stress, fatigue, and muscle soreness scores are presented in Table 4.

No significant period was reported for sleep (test = 7.96, *p* > 0.05, Kendall’s W = 0.11), stress (test = 10.23, *p* > 0.05, Kendall’s W = 0.14), or muscle soreness (test = 7.68, *p* > 0.05, Kendall’s W = 0.10) (Table 4).

For the fatigue scores, the Friedman test revealed a significant main effect (test = 11.74, *p* < 0.05, Kendall’s W = 0.16). Pairwise comparisons revealed significant increases in fatigue scores for AR compared with BR (*p* < 0.05) after N0. At AR, the fatigue scores were significantly lower after N35 compared to N0 (*p* < 0.01).

### 3.8. The Profile of Mood State

The Friedman test did not show any significant effect on total mood scores (test = 3.34, *p* > 0.05, Kendall’s W = 0.04), tension (test = 3.78, *p* > 0.05, Kendall’s W = 0.05), anger (test = 4.69, *p* > 0.05, Kendall’s W = 0.06), confusion (test = 0.77, *p* > 0.05, Kendall’s W = 0.01), depression (test = 7.74, *p* > 0.05, Kendall’s W = 0.11), fatigue (test = 9.62, *p* > 0.05, Kendall’s W = 0.13), and vigor (test = 9.04, *p* > 0.05, Kendall’s W = 0.12) (Table 5).

## 4. Discussion

The purpose of this study was to investigate the effects of a 35-min nap on physical performance during the 5mSRT; attention; feelings; mood states; and the perception of stress, muscle soreness, and fatigue before, during and after Ramadan observance in young physically active men. The main findings of the present study were as follows: (*i*) TD and BD significantly improved after N35 in comparison with N0 at BR, DR, and AR and (*ii*) attention was significantly higher after N35 in comparison with N0 at DR and AR.

During N0, findings revealed that TD and FI were not affected by Ramadan observance; this supports the results of Boukhris et al. [36], who reported that TD and FI during the 5mSRT were not significantly affected by the fasting period. As previously suggested by Boukhris et al. [36], the maintained TD during Ramadan observance could be related to the unchanged total fat intake during Ramadan. In fact, fat oxidation could assist endurance performance by mitigating the onset of fatigue via the sparing of muscle glycogen [39]. Additionally, Chtourou et al. [12] suggested that perturbation in mood states is one of the main reasons for impeded performance during Ramadan. Supporting our findings, mood state parameters, stress, sleep, and muscle soreness were unaffected by Ramadan, which may have assisted the unchanged TD and FI.

However, in contradiction with the results of Boukhris et al. [36], this study demonstrated that BD was significantly lower in DR compared to AR (120.87 m ± 2.89 m vs. 125.8 m ± 2.1 m). This result is in agreement with those of Chtourou et al. [12], who reported that performances during repeated cycling exercise and the Wingate test were affected by Ramadan fasting in young footballers. Chtourou et al. [12] explained this impairment by the fact that fatigue was higher in DR compared to BR. Similarly, RPE scores recorded after the 5mSRT were significantly increased in DR in comparison with AR. Also, feelings scores recorded after the 5mSRT were lower in DR than BR. Thus, the increased perception of fatigue and the reduced feelings state could explain high performance attainment in DR. This contradiction between the present results and those of Boukhris et al. [36] could be explained by the fact that sleep scores in the present study were negatively affected by Ramadan observance. Thus, worse sleep impacted the ability to perform at the highest intensity during the 5mSRT. Also, BD (i.e., registered in all cases for the first 30-s repetition) is possibly related to alactic energy usage or adenosine triphosphate and phosphocreatine (ATP-PCr) capacity. The decreased fractional contribution of carbohydrate may also explain the decline of BD during DR.

Attention estimated by the digit cancellation test was significantly lower at DR in comparison with BR and AR; this supports the results of Boukhris et al. [11], who reported that Ramadan observance had an adverse effect on attention. Also, supporting previous literature [4,5,11], the impaired sleep reported in DR could be related to a decrement in cognitive performance, such as an attention task. Indeed, there was a significant increase in total scores on the PSQI and the subjective sleep quality scores DR in comparison with BR, with a significant decrease of sleep duration in DR compared to BR. In addition, although total PSQI scores were higher at AR in comparison with BR, it does not indicate poor sleep quality. In fact, PSQI scores reported during Ramadan (PSQI scores = 4.64) were above 5 [40].

It was hypothesized that a 35-min nap may be beneficial for all measured parameters both outside and during Ramadan. This hypothesis was partially confirmed. Although no changes have been reported for the perception of mood states, stress, sleep, muscle soreness, and the FI during the 5mSRT, BD and HD were better after N35 compared to N0 at BR, DR, and AR. Additionally, the other parameters reached significance (N35 better than N0) only in some periods of study: attention at DR and AR, RPE at DR, feelings at AR, and fatigue estimation at AR. In contradiction, Hsouna et al. [29] did not report a significant effect of the 25-min nap opportunity on physical performance during the 5mSRT and attention. However, in support of the speculation of the study of Hsouna et al. [28], increasing the nap opportunity to 35 min allowed for significant positive effects on cognitive and short-term repetitive maximal performances. The benefits observed only after a 35-min nap and not after a 25-min nap could be related to increased inclusion of slow-wave sleep, known as a recovery period for daily metabolism [26]. Previous studies, out of Ramadan, have reported that a 35-min nap opportunity is beneficial for performance improvement and perception of fatigue reduction during the 5mSRT [26]. To the best of the authors’ knowledge, this is the first study investigating the effect of a nap opportunity during Ramadan observance on physical and cognitive performance. Although positive effects have been observed on physical performance (i.e., increase of TD and BD after N35 in the all experimental sessions) and cognitive performance (i.e., improvement of attention after N35 at DR and AR), previous studies show that napping might improve physical [24,25,26,27,28,41] and cognitive [28,41] performance. The non significant effect of the nap opportunity on some parameters may be related to duration. It is possible that, if the nap time was increased, significant differences may have been observed for a majority of the dependent variables. For example, with lower duration, Petit et al. [42] reported that a 20-min nap did not improve performance during the 30-s Wingate test after normal or 5-h phase advanced sleep. In this context, Hammouda et al. [25] concluded that better improvement in physical performance was recorded after the longer nap duration (i.e., after a 90-min compared to 20-min nap opportunity). Boukhris et al. [26] reported that, compared to 25 min and 35 min, 45 min is a better duration for improving the 5mSRT performance. Additionally, Hsouna et al. [28] concluded that the beneficial effect of a nap after a normal sleep night on the 5-jump test performance exists when the nap duration was over 35 min. It has been reported that the improvement of physical performance is associated with a reduction of subjective fatigue [26,43], feelings scores [28], and an improvement in alertness [43]. These findings are, partially, in agreement with the results of the present study, which confirmed a decrease of RPE scores after N35 at DR, an improvement of attention, and the estimated fatigue at AR.

Although the present study presented important practical recommendations, some limitations exist. First, the present study lacks an objective measurement of the night and the nap sleep (e.g., by the use of actigraphy or polysomnography). Future studies should utilize registration of the previous night’s sleep and the nap during the experimental period. In addition, a specific sleep scale relating to quality of sleep during the nap should be used for future studies. Moreover, due to a technical issue, the current study lacks control of the luminosity level during the sleeping period; this drawback should be avoided in future studies. In addition, to confirm the results of the present study, further studies should compare different nap durations outside and during Ramadan.

## 5. Conclusions

The results revealed that TD and FI during the 5mSRT were not affected by Ramadan observance. However, BD was significantly lower in DR compared to AR after N0 and N35. During N0 condition, attention decreased significantly in DR in comparison with BR and AR. BD and TD were better after N35 compared to N0 at BR, DR, and AR. A significant increase of attention after N35 in comparison with N0 at DR and AR was observed. However, no changes have been reported for the perception of mood states, stress, sleep, muscle soreness, and FI during the 5mSRT. Additionally, N35 was better than N0 for attention at DR and AR, RPE at DR, feelings at AR, and fatigue estimation at AR. We suggested that further increases of the nap duration during Ramadan would allow for significant effects of the nap in all parameters before, during, and after the fasting month.

Therefore, coaches and athletes should plan some periods of 35-min daytime naps during Ramadan observance to overcome any sleep loss and/or to improve physical and cognitive performances.

## Figures and Tables

**Table 1:** Feelings, rating of perceived exertion (RPE), and attention scores (mean ± SD) recorded before, during, and after Ramadan after no-nap (N0) and 35-min nap (N35) opportunities.

Parameters	Before Ramadan		During Ramadan		After Ramadan	
	N0	N35	N0	N35	N0	N35
Feelings Score (a.u)	0.71 ± 0.36	1.21 ± 0.37	−0.21 ± 0.23 ^¤^	0.07 ± 0.45 ^¤,#^	0.29 ± 0.24	1.07 ± 0.21 *
RPE Score (a.u)	4.59 ± 0.28 ^#^	4.22 ± 0.18	4.32 ± 0.31 ^#^	3.81 ± 0.22 *	3.92 ± 0.30	3.73 ± 0.19
Attention Score (a.u)	64.50 ± 2.52	67.64 ± 2.78	61.14 ± 3.06 ^¤,#^	67.07 ± 2.86 *	65.86 ± 2.73	75.29 ± 3.17 ^¤,^*

* Significant difference in comparison with N0; ¤ significant difference in comparison with before Ramadan; and # significant difference in comparison with after Ramadan.

**Table 2:** Dietary intake parameters (mean ± SD) recorded before, during, and after Ramadan.

Parameters	Before Ramadan	During Ramadan	After Ramadan
Energy Intake (Kcal/J)	2696 ± 142	2439 ± 113	2410 ± 145
Carbohydrates (%)	50.5 ± 1.80	45.86 ± 2.03	52.51 ± 1.15 *
Lipids (%)	35.07 ± 2.43	40 ± 2.34	34.71 ± 1.27
Proteins (%)	14 ± 2.13	12.64 ± 0.59	13 ± 0.63

* Significant difference in comparison with during Ramadan.

**Table 3:** Pittsburgh Sleep Quality Index (PSQI) parameters (mean ± SD) recorded before, during, and after Ramadan.

Parameters	Before Ramadan	During Ramadan	After Ramadan
Sleep Quality (a.u)	0.86 ± 0.23	1.93 ± 0.20 *	1.57 ± 0.23 *
Sleep Latency (min)	15.36 ± 1.92	17.57 ± 2.38	17.43 ± 1.70
Sleep Duration (h)	8 ± 0.48	6.79 ± 0.45 *	7.21 ± 0.42
Sleep Efficiency (%)	95.18 ± 2.36	93.65 ± 2.09	95.24 ± 1.83
Sleep Disturbances (a.u)	0.57 ± 0.14	0.79 ± 0.15	0.64 ± 0.13
Daytime Dysfunction (a.u)	0.21 ± 0.11	0.79 ± 0.21 *	0.29 ± 0.13
Total PSQI Scores (a.u)	3.07 ± 0.65	6.07 ± 0.68 *	4.64 ± 0.56 *^,#^

* Significant difference in comparison with before Ramadan; # significant difference in comparison with during Ramadan.

**Table 4:** Sleep, stress, fatigue, and muscle soreness scores (mean ± SD) recorded before, during, and after Ramadan after no-nap (N0) and 35-min nap (N35) opportunities.

Parameters	Before Ramadan		During Ramadan		After Ramadan	
	N0	N35	N0	N35	N0	N35
Sleep (a.u)	3.86 ± 0.47	3.43 ± 0.31	4.50 ± 0.45	4.07 ± 0.40	4.43 ± 0.31	4.00 ± 0.21
Stress (a.u)	3.43 ± 0.33	3.07 ± 0.38	2.50 ± 0.37	3.00 ± 0.42	2.71 ± 0.37	2.36 ± 0.31
Fatigue (a.u)	3.93 ± 0.30	4.00 ± 0.30	3.93 ± 0.47	3.79 ± 0.37	4.57 ± 0.27 *	3.64 ± 0.13 ^#^
Muscle Soreness (a.u)	3.36 ± 0.40	3.14 ± 0.33	2.93 ± 0.34	2.57 ± 0.25	3.21 ± 0.26	2.86 ± 0.29

* Significant difference in comparison with before Ramadan; # significant difference with N0.

**Table 5:** Profile of mood state (POMS) parameters (mean ± SD) recorded before (BR), during (DR), and after (AR) Ramadan after no-nap (N0) and 35-min nap (N35) opportunities.

Parameters	Before Ramadan		During Ramadan		After Ramadan	
	N0	N35	N0	N35	N0	N35
Total Mood Scores (a.u)	23.21 ± 9.02	16.50 ± 8.45	21.21 ± 9.36	17.07 ± 8.82	24.21 ± 7.99	15.50 ± 4.93
Tension (a.u)	9.57 ± 1.76	8.86 ± 1.56	9.57 ± 1.66	7.86 ± 1.62	9.14 ± 1.37	7.93 ± 1.21
Anger (a.u)	11.37 ± 2.58	9.07 ± 1.91	9.43 ± 2.59	8.57 ± 2.38	9.64 ± 1.95	7.93 ± 1.62
Confusion (a.u)	6.86 ± 1.14	6.36 ± 1.45	6.57 ± 1.13	6.21 ± 1.13	7 ± 1.11	6.79 ± 1.05
Depression (a.u)	8.07 ± 2.42	7.64 ± 2.39	6.79 ± 2.73	6.79 ± 2.94	9 ± 2.48	7.71 ± 1.70
Fatigue (a.u)	6.71 ± 1.56	4.93 ± 1.30	6 ± 1.62	4.71 ± 1.47	6.43 ± 1.47	3.79 ± 0.85
Vigor (a.u)	19.36 ± 1.78	20.36 ± 1.64	17.14 ± 2.04	17.07 ± 1.64	17 ± 1.82	16.07 ± 2.07
